# High Pretreatment DHEA Is Associated with Inferior Immunotherapy Response in Metastatic Non-Small Cell Lung Cancer

**DOI:** 10.3390/cancers16061152

**Published:** 2024-03-14

**Authors:** Yumeng Zhang, Lancia Darville, Stephanie Hogue, Julie E. Hallanger Johnson, Trevor Rose, Youngchul Kim, Alexis Bailey, Jhanelle E. Gray, Lary A. Robinson

**Affiliations:** 1 Department of Malignant Hematology, Moffitt Cancer Center, Tampa, FL 33612, USA; yumeng.zhang@moffitt.org; 2 Proteomics and Metabolomics, Moffitt Cancer Center, Tampa, FL 33612, USA; lancia.darville@moffitt.org; 3 Division of Cancer Epidemiology, Moffitt Cancer Center, Tampa, FL 33612, USA; stephanie.hogue@moffitt.org; 4 Department of Head and Neck-Endocrine Oncology, Moffitt Cancer Center, Tampa, FL 33612, USA; hallangerjohnson.julie@mayo.edu; 5 Department of Radiology, Moffitt Cancer Center, Tampa, FL 33612, USA; trevor.rose@moffitt.org; 6 Department of Biostatistics and Bioinformatics, Moffitt Cancer Center, Tampa, FL 33612, USA; youngchul.kim@moffitt.org; 7 Department of Thoracic Oncology, Moffitt Cancer Center, Tampa, FL 33612, USA; alexis.bailey@moffitt.org (A.B.); jhanelle.gray@moffitt.org (J.E.G.)

**Keywords:** androgens, sex hormones, DHEA, immune checkpoint inhibitors, non-small cell lung cancer

## Abstract

**Simple Summary:**

In recent years, treatments that help our immune system fight cancer, known as immune checkpoint inhibitors (ICIs), have made significant strides in treating lung cancer. However, not all patients respond the same way to these therapies. Our study seeks to understand if levels of a hormone called DHEA in the blood before treatment can predict how well patients with advanced lung cancer will respond to ICI therapies. Patients with lower levels of DHEA before treatment tended to do better, experiencing longer periods without cancer progression and living longer overall compared to those with higher DHEA levels. The findings could open new doors for improving treatment strategies and outcomes for people with lung cancer.

**Abstract:**

*Background:* Sex difference in the immune response may influence patients’ response to immune checkpoint inhibitors (ICIs). We conducted a prospective observation study to determine the correlation between pretreatment sex hormone levels and response to ICIs in metastatic non-small cell lung cancer (NSCLC). *Method:* Pretreatment plasma samples from 61 patients with newly diagnosed NSCLC prior to ICI therapy were collected. Six sex hormone levels [pyrazole triol, 17 β-estradiol, 5-androstenediol, 3β-androstenediol, dehydroepiandrosterone (DHEA), and S-equol] were measured using liquid chromatography coupled to high-resolution mass spectrometry (LC-HRMS). Overall survival (OS) and progression-free survival (PFS) were compared between the high- and low-level groups in the whole cohort. *Result:* Among the six sex hormones measured, DHEA levels were significantly higher among patients without clinical benefits in the discovery cohort; the remaining sex hormones did not differ significantly. In the whole cohort, median PFS was 22 months for patients with low DHEA levels vs. 3.8 months for those with high DHEA [hazard ratio, 14.23 (95% CI, 4.7–43); *p* < 0.001]. A significant association was also observed for OS [hazard ratio, 8.2 (95% CI, 2.89–23.35); *p* < 0.0001]. *Conclusions:* High pretreatment plasma DHEA levels were associated with poor clinical outcomes for patients with metastatic NSCLC treated with ICIs.

## 1. Introduction

Immune checkpoint inhibitors (ICIs), such as anti-programmed cell death protein 1/programmed cell death protein-ligand 1 (PD1/PD-L1) therapy, have changed the treatment paradigm for solid tumors. ICIs work by interrupting the inhibitory signals in T-cell activation, augmenting a more robust immune response from tumor-reactive T cells [1]. Sex differences in immune response are a known phenomenon. Generally, females are more prone to developing autoimmune disease but less susceptible to infection and malignancy [2]. Additionally, patients’ sex may influence their therapeutic response to ICIs.

Previous pan-cancer meta-analyses have shown conflicting results on whether there is an association between sex and the efficacy of ICIs [3,4,5]. Ye and associates evaluated multiple cancer types to address the heterogeneity in the results of clinical trials assessing sex-related differences in ICI efficacy [6]. Female patients undergoing anti-PD-1/PD-L1 therapy for non-small cell lung cancer (NSCLC) and esophageal cancer tended to have longer overall survival (OS) than males. In contrast, males treated for colorectal cancer or glioblastoma tended to have longer OS than females [6]. In a meta-analysis, Conforti, et al. confirmed that women with NSCLC derived statistically significant greater benefits from adding ICIs to chemotherapy regimens than males [4]. Taken together, these studies suggest that sex differences in response to ICIs are dependent on the cancer type but remain somewhat unclear. The causes of these potential sex differences in treatment effectiveness are unknown at this time but are hypothesized to be related to genetic content, such as Y chromosomes, and sex hormones.

Sex hormones are critical in regulating the immune system and response. Estrogen and androgen receptors are expressed in various immune cells, including T cells, B cells, macrophages, and dendritic cells [2,7]. For example, dehydroepiandrosterone (DHEA) and 5-androstenediol primarily bind to androgen receptors, whereas pyrazole triol (PPT), 17β-estradiol (E2), 3β-androstenediol (3β-diol), and S-equol primarily bind to estrogen receptors. Sex hormones modulate the immune response by stimulating different cytokine profiles, changing antibody production, and affecting T-cell activation and natural killer cell cytotoxicity [7]. For example, DHEA, often characterized as supporting the immune system, plays a complex interaction with different immune components, including its role in upregulating glucocorticoid receptor beta expression [8]. However, the exact mechanisms by which sex hormones influence immune response and affect outcomes for cancers like NSCLC are complex and still being explored.

Preliminary evidence, such as the work by Guan et al., has begun to shed light on the complex interplay between sex hormones and ICI responsiveness. Specifically, their investigation into androgen deprivation therapy highlighted its potential to enhance the effectiveness of ICIs in prostate cancer by modulating androgen receptor activity and improving T-cell function [9,10]. These insights suggest the significant influence of sex hormones on treatment outcomes in prostate cancer. Despite growing evidence, the direct correlation between pretreatment sex hormone levels and ICI response, particularly in NSCLC, remains largely unexplored. Our prospective observational cohort study aims to fill this gap by examining the association between pretreatment plasma levels of specific sex hormones, including PPT, E2, 5-androstenediol, 3β-diol, DHEA, and S-equol, and clinical outcomes in patients with metastatic NSCLC undergoing ICI treatment.

## 2. Materials and Methods

### 2.1. Study Design, Setting, and Participants

This study used plasma samples collected from a prospective observational cohort study, which involved patients treated for metastatic NSCLC at H. Lee Moffitt Cancer Center and Research Institute (Tampa, FL, USA) between 1 May 2016 and 1 August 2018. The data cut-off was 1 September 2019. We consecutively enrolled adult patients with histologically confirmed newly diagnosed metastatic NSCLC who were scheduled to undergo ICI therapy with or without chemotherapy. The treating medical oncologists determined the choice of immunotherapy and chemotherapy. Patients receiving hormone replacement or deprivation therapies were excluded. Informed consent was obtained from all patients. This study was approved by the Advarra Institutional Review Board (MCC 18611, PRO00017235).

A subset of the overall cohort, termed the discovery cohort, included the first 16 patients (8 men and 8 women) in the study. The discovery cohort was used to develop a hypothesis, identify a potential marker hormone, define sex-specific cut-off points, and estimate the sample size for the whole cohort. Sixteen patients were divided into two groups based on clinical benefit status. Clinical benefit status was defined as complete response (CR), partial response (PR), or stable disease (SD) for at least 12 months.

### 2.2. Data Collection

Baseline data extraction included demographic characteristics, smoking history, Eastern Cooperative Oncology Group (ECOG) performance status, body mass index, histology, PD-L1 expression, somatic mutation status, medical comorbidities, prior systemic therapies (adjuvant or neoadjuvant) received within 12 months, pretreatment laboratory data, type of ICI received, and menopausal status (for females). Medical records were reviewed monthly until tumor progression or death. Response to immunotherapy and tumor progression were determined by treating physicians. PET/CT or CT scans were independently reviewed and confirmed by our study’s radiologist (T.R.) based on RECIST v1.1 criteria [11]. We determined that the best clinical indicator of ICI effectiveness was either response (CR or PR) or SD for at least 12 months [12,13,14]. In our study, we defined clinical benefit as including stable disease (SD) for at least 12 months, aiming to identify a patient subgroup that demonstrated significant benefit from immunotherapy despite not meeting RECIST criteria for response. This approach acknowledges the clinical relevance of maintaining disease stability over this period in aggressive lung cancer treated with ICIs. We chose a 12-month cutoff for this definition based on the observation that the median duration of response with combination chemotherapy and immunotherapy (IO) observed in the KEYNOTE-189 study was 11.2 months and 7.7 months in the KEYNOTE-407 study, with KEYNOTE-024′s IO alone group not reaching a median duration [15,16,17]. Thus, patients who do not show disease progression or death beyond 12 months are considered to derive substantial clinical benefits. This comparison underlines our rationale for the 12-month threshold, situating it within the context of existing clinical benchmarks.

### 2.3. Sample Collection

Blood samples were collected within 48 h before the initiation of immunotherapy. At baseline, 10 mL of blood was collected in ethylenediaminetetraacetic acid (EDTA) tubes. To isolate five 1 mL aliquots of plasma, samples were centrifuged at 1500× *g* to 3000× *g* at 4 °C for 15 min. After snap freezing, these aliquots were stored at −80 °C for further analyses.

### 2.4. Quantification of Sex Hormones in Plasma

The reference protocol [18] was modified and optimized for sample preparation and quantitation of estrogens and androgens in plasma samples. Briefly, a mixture containing 2 ng of 13C6-labeled E2 internal standard and 10 ng of deuterium-labeled DHEA (D6-DHEA) and D3-5-androstenediol were added to each 0.5 mL plasma sample. Steroids were hydrolyzed overnight at 37 °C using β-glucuronidase/sulfatase from Helix pomatia (1000 U) in 0.15 M sodium acetate buffer (pH 4.6) containing 2.5 mg L-ascorbic acid to deglucuronidate and desulfate the steroids in the plasma. Steroids were extracted by adding the hydrolyzed sample to a solid-support liquid extraction cartridge (3 mL, unbuffered) and eluted with 3 mL of dichloromethane three times for a total of 9 mL of eluent, and then they were dried under a stream of nitrogen. The estrogens (S-equol, E2, and PPT) in the extract were derivatized with 3 mg/mL of 1-methylimidazole-2-sulfonyl chloride (MIS) in anhydrous acetone at 60 °C for 15 min. Derivatized samples were dried under a stream of nitrogen and re-suspended in 50 μL of loading solvent (30% methanol containing 0.1% formic acid) for ultra-high-performance liquid chromatography–high-resolution mass spectrometry (UHPLC-HRMS) analysis.

An assay targeting PPT, E2, 5-androstenediol, 3β-diol, DHEA, and S-equol using UHPLC-HRMS was performed using a Vanquish UHPLC system and Q Exactive Focus mass spectrometer (Thermo Fisher Scientific; Waltham, MA, USA). A total of 25 μL of sample was injected onto the column. Separation was performed on a C18 reversed phase column (2.1 mm inner diameter × 100 mm length, with 2 μm particle size) maintained at 35 °C with a 0.250 mL/min flow rate. Solvent A was composed of high-performance liquid chromatography (HPLC)-grade water containing 0.1% formic acid; solvent B was composed of methanol containing 0.1% FA. The assay was performed as follows: a linear gradient was programmed from 30% to 90% B over 14 min with a flow rate of 0.250 mL/min; the gradient was maintained at 90% B for 2 min to wash the column; re-equilibration was performed for 3 min at a flow rate of 0.45 mL/min; and the gradient was returned to the original flow rate for 2 min. The assay took a total of 21 min for each experiment. The Q Exactive Focus mass spectrometer was equipped with an atmospheric pressure chemical ionization probe and operated using the following parameters for ionization in positive mode: spray voltage of 4.0 kV, 70 000 resolution, 100 ms maximum injection time, and scan range from *m*/*z* 240 to *m*/*z* 900.

Calibration standards prepared with charcoal-stripped human plasma (CSHP) were extracted, analyzed, and compared for overall sensitivity; they were analyzed in duplicate. Normal human plasma (P9523 [MilliporeSigma, Burlington, MA, USA]) was used to evaluate the assay’s performance. Calibration standards were prepared by adding 2 ng of 13C6-labeled E2 internal standard, as well as 10 ng of D6-DHEA and D3-5-androstenediol to each CSHP. Additionally, varying amounts of a working estrogen standard solution (ranging from 3 to 3000 ng/mL for DHEA, 5-androstenediol, and 3α-diol and from 0.006 to 400 ng/mL for PPT, E2, and S-equol) were also added to CSHP. Peak heights of the plasma endogenous metabolites and their respective internal standards were determined using Skyline [19] (Version 20.1.0.31, University of Washington, Seattle, WA, USA) to extract peak heights with a mass tolerance of 5 ppm. High-resolution mass spectrometry quantification was accomplished using the peak at the *m*/*z* of the MIS-derivatized estrogens and the *m*/*z* of the androgens with loss of water. Relative quantification was determined for S-equol and PPT using 13C6-labeled E2 and 3β-diol using D3-5-androstenediol.

### 2.5. Statistical Analyses

In the discovery cohort, pretreatment sex hormone levels (i.e., PPT, E2, 5-androstenediol, 3β-diol, S-equol, and DHEA) were compared between the Clinical-Benefit and No-Clinical-Benefit groups using the Mann–Whitney test and the Holm-Šídák method was used to correct for multiple comparisons with an alpha value of 0.1. Once a potential biomarker was identified, we used the receiving operating characteristics (ROC) curve to determine sex-specific cut-off points and dichotomized those with high vs. low levels of circulating sex hormones [20].

The overall study’s sample size was determined based on the DHEA level from our discovery cohort because we observed a differential abundance of DHEA between the Clinical-Benefit and No-Clinical-Benefit groups by sex. Therefore, a power analysis was used to detect the interaction effect of sex and clinical benefit on DHEA. A total sample size of at least 32 was needed to achieve 91% power to detect a significant interaction effect with an overall type 1 error rate of 5%.

We compared the clinical characteristics of the patients with high and low DHEA levels. Categorical variables were presented as percentages and compared using the chi-square or Fisher exact tests. Continuous variables were presented as medians and interquartile ranges and compared using the Kruskal–Wallis test. Swimmer’s plots were used to present individual patients’ clinical responses to immunotherapy, separated by androgen level.

We used univariate and multivariate Cox proportional hazards models to measure hazard ratios (HRs) for PFS, which was defined as the time from first ICI receipt to disease progression or death from any cause, and for OS, which was defined as the time from first ICI receipt to death from any cause. PFS and OS comparisons were also evaluated using Kaplan–Meier estimates and log-rank tests. All *p* values were two-sided. All statistical analyses were performed in SPSS Statistics 26 (IBM; Armonk, NY, USA), and graphs were made using Prism GraphPad 9.3.1 (GraphPad Software, Inc., San Diego, CA, USA).

## 3. Results

### 3.1. Patient Demographics and Clinical Characteristics of the Whole Cohort

Between 1 May 2016 and 1 August 2018, a total of 136 patients were screened and 61 patients had baseline plasma samples available for analysis. At a median follow-up of 20 months, 27 patients (44%) had died.

The median age of the whole cohort was 67 years (range 44–85 years), and 31 patients (50.8%) were women, all post-menopausal (Table 1). Fifty-four patients (88%) were white, and eight (13.1%) had never smoked. Twenty-seven patients (44%) received systemic chemotherapy within the past 12 months in either neoadjuvant or adjuvant settings, but none had received ICIs before the study. All patients received either PD-1 or PD-L1 inhibitors, and six patients (10%) received investigational therapy (interleukin 2) together with ICIs and chemotherapy. Among 51 patients with available PD-L1 statuses, 33 (65%) had PD-L1 expression ≥1%. The Clinical-Benefit group had fewer patients with diabetes mellitus (Table 1).

### 3.2. Plasma Sex Hormone Levels in the Discovery Cohort

**Figure 1:**
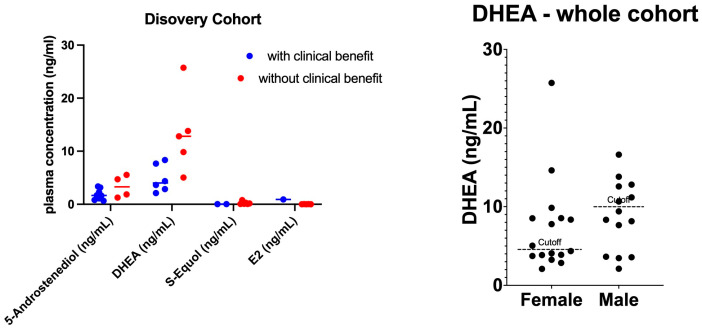
Plasma sex hormone concentration for Clinical-Benefit and No-Clinical-Benefit groups. (**Left)**: Scatter plot of plasma sex hormone levels. Dots represent the plasma hormone level for each patient, and bars represent the median. (**Right**): DHEA value of the whole cohort. Dotted line annotates the selected cutoff value. Abbreviations: DHEA, dehydroepiandrosterone; E2, 17 β-estradiol.

**Figure 2:**
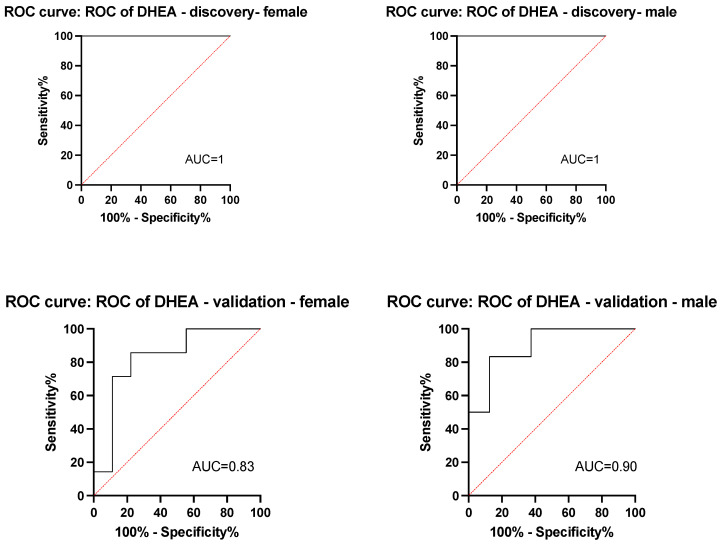
Receiver operating characteristics curve of DHEA. The *x*-axis represents sensitivity, %, and the *y*-axis represents specificity, %. AUC summarizes the performance of the classifier over all possible discrimination thresholds. An AUC of 1 indicates perfect performance, and 0.5 indicates performance that is no better than random chance. The top panel shows the ROC curve for the discovery cohort, and the bottom panel shows the ROC curve for the validation cohort. Abbreviations: AUC = area under the ROC curve; DHEA = dehydroepiandrosterone; ROC = receiver operating characteristics.

We measured DHEA, 5-androstenediol, S-equol, PPT, and E2 concentrations among the pretreatment plasma samples of patients in the discovery cohort. Only one patient had a detectable PPT level; therefore, the analysis did not include PPT. Plasma DHEA levels were significantly higher among patients who experienced no clinical benefit (median, 4 ng/mL in the Clinical-Benefit group vs. 12.8 ng/mL in the No-Clinical-Benefit group; *p* = 0.017) (Figure 1, left). The levels of the remaining sex hormones (5-androstenediol, S-equol, and E2) did not differ significantly. Based on ROC analyses, DHEA cut-off points were selected as 4.7 ng/mL for women [sensitivity, 100% (95% CI, 43.85% to 100%); specificity, 100% (95% CI, 17.77–100%)] and 10.03 ng/mL for men [sensitivity, 100% (95% CI, 17.77–100%); specificity, 100% (95% CI, 51–100%)] (Figure 2, top panel). The selected cut-off value for DHEA was similar to the median of the whole cohort. (Figure 1, right).

### 3.3. Patients with High DHEA Levels Had Poor Clinical Outcomes in the Whole Cohort

**Figure 3:**
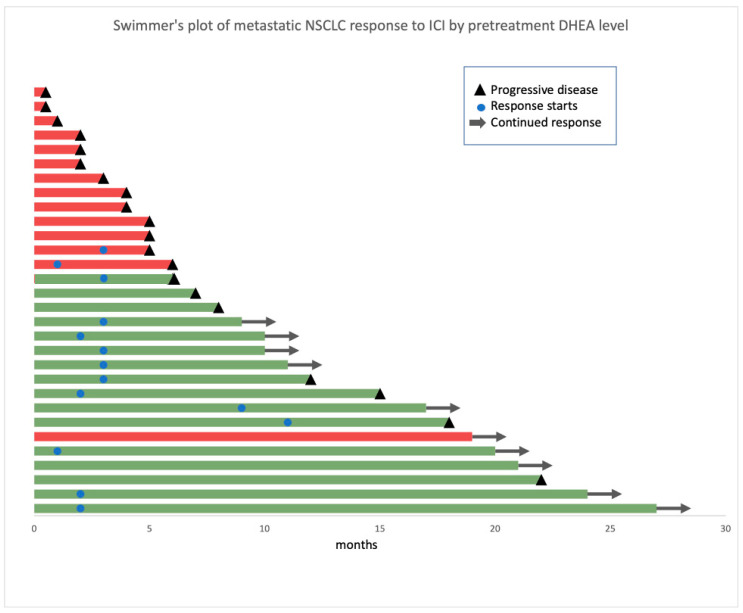
Swimmer’s plot of clinical response by DHEA level. Patients are color-labeled based on their DHEA level and arranged based on their duration of therapy. The *x*-axis represents patients’ duration of therapy in months. A red bar represents individuals with high DHEA, and a green bar represents individuals with low DHEA. Abbreviations: DHEA, dehydroepiandrosterone.

In the whole cohort, 30 patients had no measurable DHEA level. Among 30 patients with measurable DHEA levels, 16 had low DHEA levels. The clinical characteristics of patients with low and high DHEA levels are summarized in Table 1. The groups had no significant differences in clinical characteristics, laboratory values, or co-mutational status. PD-L1 expression was positive in 69% and 77% of patients with low and high DHEA levels, respectively (*p* = 0.58). Only one patient with a low DHEA level had PD-L1 expression ≥90%. In total, 31% of patients with low DHEA levels and 43% with high DHEA levels received ICI only; the remainder received ICI and chemotherapy. In the Swimmer’s plot of clinical response by DHEA level, most patients with low DHEA levels derived clinical benefit from ICI therapies (Figure 3). The only patient in the high-DHEA group who experienced significant clinical benefit had a very low volume of disease at baseline and received both chemotherapy and an ICI.

For patients with measurable DHEA, the median PFS was 8.2 months, and the median OS was 16.9 months. The median PFS and OS were 8.6 months and 18.4 months, respectively, for patients with unmeasured DHEA levels, similar to those with measurable DHEA.

**Figure 4:**
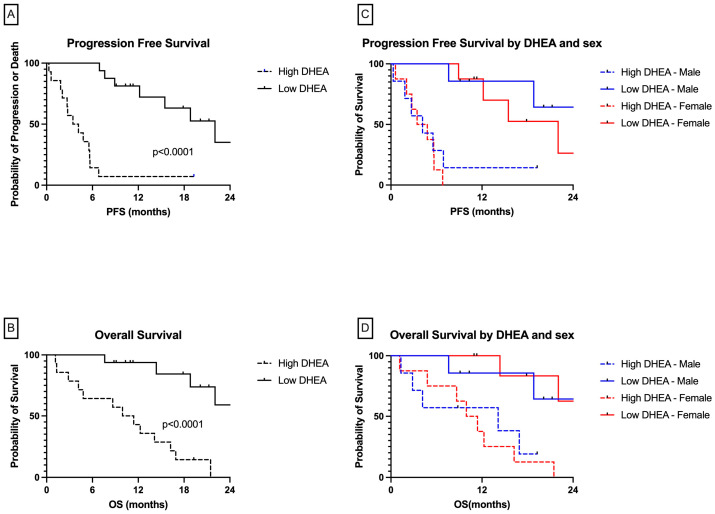
Clinical outcomes based on pretreatment DHEA level for (**A**) progression-free survival by DHEA, (**B**) overall survival by DHEA, (**C**) PFS by DHEA and sex, and (**D**) OS by DHEA and sex. Abbreviations: DHEA, dehydroepiandrosterone; OS, overall survival; PFS, progression-free survival.

When examining the survival outcomes by DHEA level, we observed a statistically shorter PFS among patients with high DHEA than those with low DHEA, with an HR of 14.23 (95% CI, 4.7–43; *p* < 0.001) (Figure 4A). The median PFS was 22 months for patients with low DHEA vs. 3.8 months for patients with high DHEA. Similarly, OS analysis showed a significant association, with an HR of 8.2 (95% CI, 2.89–23.35; *p* < 0.0001) (Figure 4B). The median OS for patients with low DHEA was not reached, whereas the median OS for patients with high DHEA was 10.67 months. This association of PFS and OS with DHEA level was seen in both males and females. (Figure 4C,D) The median PFS for male patients with low and high DHEA was not reached and 4.2 months, respectively; the median PFS for female patients with low and high DHEA was 22 months and 4.1 months, respectively. The median OS for male patients with low and high DHEA was not reached and was 14 months, respectively; the median OS for the female patients with low and high DHEA was not reached and was 10 months, respectively.

The Cox proportional hazards model also confirmed this observation. A high DHEA level was associated with shorter PFS and OS in univariate analyses. After adjusting for age, sex, BMI, smoking status, and diabetes mellitus status, DHEA remained a significant predictor of survival in the multivariate analyses (Table 2).

## 4. Discussion

Our study examined sex hormone levels among newly diagnosed metastatic NSCLC patients before initiating ICI therapy. We report that patients with low DHEA levels derived more clinical benefit from ICI therapy than those with high DHEA levels. The median PFS was 18 months longer for those with low DHEA levels than those with high DHEA levels, and patients with low DHEA levels also had significantly longer OS in both male and female patients. Together, these results support the hypothesis that DHEA could play a role in the immune system’s response to ICI therapy in the upfront setting for metastatic NSCLC and suggest a rationale to explore potential immune checkpoint and androgen receptor dual blockade in NSCLC. To our knowledge, this is the first study to examine the interaction between DHEA levels and ICI response in NSCLC.

DHEA is a naturally occurring steroid hormone produced by the adrenal glands that is the prohormone to testosterone. Though its function might overlap with testosterone as a sex hormone, this understudied sex hormone may exert unique effects. DHEA levels are affected by genetic factors, such as sex, post-menopausal status, age, and environmental factors, such as flavonoid intake and glucose intake [21,22,23]. Consistent with prior results, the circulating DHEA levels are higher in men than post-menopausal women in our study [21,22]. Most current research focuses on the correlation between DHEA levels, cardiovascular health, and insulin resistance. Low DHEA levels correlated with the development of heart failure and pulmonary arterial hypertension [22,24]. Therefore, DHEA has been marketed as a dietary supplement to prevent the effects of aging and improve cardiovascular health. However, no human trials have been conducted to evaluate such claims [25]. Unlike DHEA-S, its sulfate form, no cut-off point is reported in the literature for DHEA levels. Therefore, we defined the sex-specific cut-off points using the discovery cohort. The measured DHEA level in the study is consistent with the previously reported value [21].

We hypothesize that DHEA can modulate immune systems through the activation of androgen receptors. Several preclinical studies have confirmed the immune-suppressive role of androgens through androgen receptors expressed in various innate and adaptive immune cells, such as neutrophils, macrophages, dendritic cells, B cells, and T cells [7,26,27,28,29,30]. In murine models, androgen deprivation augments T-cell activities, especially CD8+ T cells, and enhances T-cell response to T-cell receptor- and CD28-mediated co-stimulation [30,31]. Castration of male mice enhances the efficacy of antigen presentation, whereas androgen-treated female mice experience decreased efficacy of antigen presentation [32]. Androgen treatment enhances Th2 response by increasing interleukin 10 (IL-10) production in murine CD4+ T cells [33].

Recently, Guan et al. evaluated the effect of androgen deprivation therapy on tumor-infiltrating T cells and its interaction with ICIs in patients with prostate cancer [9,30,31]. They demonstrated that androgen receptors repress interferon-gamma (IFNγ) function in mice and that androgen receptor inhibition significantly improved cytotoxic T-cell function. Dual blockade of androgen receptors and PD-L1 led to significant tumor regression, which is promising for prostate cancer treatment [9]. In line with these results, Roberts et al. conducted a phase 1 clinical trial combining anti-PD-1 therapy and androgen deprivation therapy for melanoma patients previously treated with anti-PD-1 therapy. Among 14 patients who previously experienced disease progression during anti-PD-1 therapy, androgen deprivation therapy resulted in a disease control rate of 42.8% [10]. Together, these findings support that combining androgen receptor blockade with ICIs is a promising future cancer research direction. This could be further explored in patients with NSCLC who progressed or relapsed on first-line ICIs, as this population has limited therapeutic options.

Instead of androgen receptor activity, the disparity in plasma DHEA levels observed in our patient cohort may potentially illustrate an underlying cortisol imbalance. Ectopic sources, such as malignancy, could drive both cortisol and DHEA secretion [34]. Consequently, elevated DHEA levels in these patients might serve as a surrogate marker for increased cortisol levels, which could in turn negatively impact the efficacy of ICIs in NSCLC. This theory remains speculative within the context of our study, as cortisol measurements were not included in our patient evaluations,

In addition to DHEA, sex itself could be a prognostic factor instead of a predictive factor in clinical outcomes in NSCLC. Data from the Korean Association for Lung Cancer Registry showed that male patients with NSCLC had poorer prognoses compared to females after adjusting for clinical variables, and the difference was consistent across stages of diseases [35]. Another comprehensive analysis using Swedish national population-based registries described the sex difference in NSCLC outcomes. It highlighted that the clinicopathological characteristics of diagnoses varied between men and women, possibly influencing diagnostic and treatment approaches and survival [36]. In line with these results, our study acknowledges the potential impact of sex as a prognostic factor in NSCLC. This consideration is particularly relevant in our investigation into DHEA levels and their relationship with ICI outcomes in NSCLC.

### Limitations

A notable limitation of our study is that 31 patients in the whole cohort did not have measurable plasma DHEA levels. DHEA levels may have been undetectable because DHEA has a short half-life, about 1–3 h, in the bloodstream [37]. Consequently, these patients were excluded from the evaluation as it was difficult to determine whether their DHEA level was lower than the detectable range’s lower limit or resulted from the timing of the measurement. In hindsight, DHEA-S may be a more optimal biomarker due to its more stable structure and extended 10–20 h circulation time [38]. Another limitation of the study was the sample size of 61 patients, though a focused group could limit the generalizability of our findings. This size of the study reflects the pilot nature of our research. However, a significant differential effect of DHEA was still detected with the limited sample size, suggesting a large effect size. The third limitation is that we could not evaluate whether DHEA is a predictive or prognostic biomarker because we did not include a patient cohort not treated with ICI.

Despite its limitations, our study explored the interesting and surprising interaction between DHEA and ICIs. However, further validation is needed with a larger prospective cohort of NSCLC patients. Finally, fundamental scientific research is needed to explore the interaction between DHEA and immune response in NSCLC to determine whether DHEA directly promotes tumor growth or inhibits immune response in ICIs.

## 5. Conclusions

Our study supports the fact that pretreatment plasma DHEA levels were negatively associated with the response to ICI therapy among patients with NSCLC. High DHEA levels were associated with poor clinical outcomes among newly diagnosed metastatic NSCLC patients treated with upfront ICIs.

Further validation with a larger independent cohort of NSCLC patients is needed. If this negative effect of DHEA level on ICIs is substantiated, then employing androgen receptor blockade during ICI therapy in NSCLC may be a rational adjunct to improve clinical response.

## Figures and Tables

**Table 1:** Patient demographics and clinical characteristics of 61 enrolled patients based on clinical benefit and DHEA level.

Characteristic	Clinical Benefit Status			DHEA Level		
	Clinical Benefit (N = 33)	No Clinical Benefit (N = 28)	*p* Value	Low DHEA (N = 16)	High DHEA (N = 14)	*p* Value
Age at diagnosis, years, median [range]	67 [44, 85]	67.5 [51, 83]	0.89	66 (54, 75)	67 (48, 83)	0.96
Male sex, No. (%)	15 (45.5)	15 (53.6)	0.527	8 (50)	6 (42.9)	0.73
Race, No. (%)			0.074			0.99
White	31 (94)	23 (82)		14 (87.5)	12 (85.7)	
Black	1 (3)	3 (11)		2 (12.5)	2 (14.3)	
BMI at time of treatment, median [95% CI]	26.1 [18.7, 36.9]	23.8 [16.9, 46.9]	0.36	26 (20, 37)	26 (20, 40)	0.14
Smoking status, No. (%)			0.803			0.71
Current	8 (24.2)	9 (32.1)		5 (31.3)	5 (35.7)	
Former	21 (63.6)	15 (53.6)		9 (56.3)	6 (42.9)	
Never	4 (12.1)	4 (14.3)		2 (12.5)	3 (21.4)	
ECOG performance status, No. (%)			0.353			1.0
0	5 (15.2)	2 (7.1)		2 (12.5)	1 (7.1)	
1	28 (84.8)	25 (89.3)		14 (87.5)	13 (92.9)	
Histology, No. (%)			0.529			0.31
Non-squamous	29 (88)	24 (86)		14 (87.5)	11 (21.4)	
Squamous	4 (12)	4 (14)		2 (12.5)	3 (64.3)	
Neutrophil-to-lymphocyte ratio, median [95% CI]	4.2 [1.8, 14.8]	4.9 [1.6, 13.7]	0.85	4.2 (2.6, 10.1)	6.4 (2.5, 13.7)	0.38
Platelet-to-lymphocyte ratio, median [95% CI]	245.6 [62.2, 1104.2]	229.9 [105.7, 796.2]	0.81	239 (137, 523)	262 (146, 1068)	0.46
Comorbidities, No. (%)						
COPD	12 (36)	16 (57)	0.11	4 (25)	8 (57.1)	0.14
HLD	22 (66.7)	17 (60.7)	0.63	12 (75)	7 (50)	0.26
MI/heart failure	3 (9.1)	6 (21.4)	0.18	1 (6.3)	4 (28.6)	0.16
DM	4 (12.1)	10 (36)	0.038	4 (25)	5 (35.7)	0.69
Prior chemotherapy, No. (%)	12 (36)	15 (54)	0.406	9 (56.3)	7 (50)	1.0
PD-L1 positive (>1%), No. positive/No. evaluable (%)	20/27 (74)	13/24 (54)	0.84	9/13 (0.69)	10/13 (0.77)	0.58
Other mutations, No. positive/No. evaluable (%)						
*ALK* fusion	1/19	1/20	0.93	1/11	0/8	0.4
*EGFR*	5/24	2/31	0.16	0/15	1/12	0.4
*KRAS*	6/19	7/31	0.07	5/14	2/10	0.39
*NRAS*	0/14	0/15	0.72	0/7	0/6	0.96
*TP53*	6/11	6/15	0.95	2/7	3/5	0.51

Abbreviations: BMI = body mass index; CNS = central nervous system; COPD = chronic obstructive pulmonary disease; DHEA = dehydroepiandrosterone; DM = diabetes mellitus; ECOG = Eastern Cooperative Oncology Group; HLD = hyperlipidemia; MI = myocardial infarction.

**Table 2:** Univariate and multivariate analysis of clinical factors affecting progression-free survival and overall survival in patients with metastatic non-small cell lung cancer undergoing immune checkpoint inhibitor therapy.

Clinical Factors	Progression-Free Survival			Overall Survival		
	No. Progression/No. Cases	Univariable HR (95% CI)	Multivariable HR (95% CI)	No. Death/No. Cases	Univariable HR (95% CI)	Multivariable HR (95% CI)
Age	45/61	1.01 (0.98–1.05)	1.03 (1.97, 1.08)	34/61	0.98 (0.94, 1.02)	0.97 (0.92, 1.04)
Sex						
Male	21/30	1	1	17/30	1	1
Female	24/31	0.85 (0.47, 1.53)	0.68 (0.23, 2.02)	17/31	1.39 (0.70, 2.73)	1.14 (0.33, 3.83)
*p* value for trend		0.58	0.48		0.35	0.36
BMI	45/61	1.00 (0.95, 1.06)	1.02 (0.92, 1.11)	34/61	0.98 (0.93, 1.05)	0.96 (0.88, 1.05)
Smoking status						
Current	13/17	1	1	12/17	1	1
Former	27/36	0.92 (0.47, 1.79)	0.25 (0.037, 1.72)	18/36	0.58 (0.28, 1.20)	0.52 (0.047, 5.62)
Never	5/8	0.84 (0.30, 2.36)	0.20 (0.018, 2.12)	4/8	0.67 (0.21, 2.07)	0.26 (0.017, 4.09)
*p* value for trend		0.94	0.58		0.33	0.43
Medical comorbidities						
DM present	10/14	1.74 (0.85, 3.59)	1.3 (0.38, 4.25)	10/14	2.63 (1.21, 5.71)	1.9 (0.49, 7.47)
DM absent	35/47	1	1	24/47	1	1
*p* value for trend		0.13	0.70		0.015	0.31
DHEA						
High	13/14	9.22 (3.23–26.31)	10.63 (3.4, 32.96)	13/14	8.75 (2.45,30.3)	11.59 (2.99, 44.9)
Low	7/16	1	1	4/16	1	1
*p* value for trend		<0.001	<0.001		<0.001	<0.001

Abbreviations: BMI = body mass index; CI = confidence interval; DHEA = dehydroepiandrosterone; DM = diabetes mellitus; HR = hazard ratio; No. = number. sex-specific cut-off points for DHEA is 10.03 ng/mL for male and 4.7 ng/mL for female.

## Data Availability

The data that support the findings of this study are available from the corresponding author upon reasonable request.
